# Video-based physiotherapy assessment of knee osteoarthritis in patients with knee pain: a validity and reliability pilot study

**DOI:** 10.1186/s12891-025-09201-x

**Published:** 2025-09-23

**Authors:** Christin Heina, Susanne Beischer, Chan-Mei Ho-Henriksson, Elvira Lange

**Affiliations:** 1 Unit of Physiotherapy, Department of Health and Rehabilitation, Institute of Neuroscience and Physiology, Sahlgrenska Academy, University of Gothenburg, Box 455, Gothenburg, SE-405 30 Sweden; 2 Sportrehab Sports Medicine Clinic, Gothenburg, Sweden; 3 Närhälsan Lidköping Rehabilitation Unit, Primary Care Rehabilitation, Region Västra Götaland, Lidköping, Sweden; 4 Research, Education, Development and Innovation, Primary Health Care, Region Västra Götaland, Göteborg, Sweden; 5 Department of General Practice, Institute of Medicine at the Sahlgrenska Academy, University of Gothenburg, Gothenburg, Sweden

**Keywords:** Digital, E-health, Osteoarthritis, Reliability, Validity, Video-based assessment

## Abstract

**Background:**

Digital health technologies are advancing rapidly, with an increasing number of physiotherapists favoring real-time, video-based platforms over telephone-based modalities. Osteoarthritis affects an estimated 595 million individuals worldwide, with approximately 62% of cases involving the knee joint. However, evidence regarding the validity and reliability of video-based assessments for knee osteoarthritis (KOA) remains scarce. The purpose of this pilot study was to explore the feasibility of video-based physiotherapy assessment of KOA in patients with knee pain. Additionally, we aimed to provide preliminary data on its concurrent validity and interrater reliability compared with conventional face-to-face assessment.

**Methods:**

A cross-sectional validity and reliability pilot study was conducted in June 2024. Participants were recruited through public advertisements. Eligible individuals were aged 45 years or older and reported knee pain. Each participant underwent both a real-time video-based physiotherapy assessment and a conventional, face-to-face assessment. The video-based assessments were recorded for later analysis. Concurrent validity was examined by determining the exact or potential agreement between the video-based and face-to-face assessments. Interrater reliability was evaluated by comparing the live video-based assessments with those obtained from the recorded video-based assessments.

**Results:**

For concurrent validity, exact agreement was observed in 28 of 35 cases (80%; κ = 0.35), indicating fair agreement. Potential agreement was achieved in 33 of 35 cases (94%; κ = 0.64), indicating substantial agreement. Interrater reliability demonstrated exact agreement in 25 of 29 cases (86%; κ = 0.52), corresponding to moderate agreement. Potential agreement for interrater reliability was observed in 27 of 29 cases (93%; κ = 0.63), corresponding to substantial agreement.

**Conclusions:**

Video-based physiotherapy assessment appears feasible and may provide preliminary indications of validity for diagnosing KOA in individuals with nontraumatic knee pain. The results suggest acceptable interrater agreement and highlight the need for more standardized digital assessment protocols to ensure consistent and reliable use in clinical practice.

**Trial registration:**

ISRCTN Registry (ISRCTN41057250), 09/05/2025. Retrospectively registered. Prospectively registered in FoU in VGR (researchweb.org) 282608, Date of registration 26/03/2024.

**Supplementary Information:**

The online version contains supplementary material available at 10.1186/s12891-025-09201-x.

## Background

The World Health Organization (WHO) has been actively engaged in developing e-health initiatives and strategies for their implementation in healthcare since 2005 [1]. Over time, e-health has evolved into the broader concept of digital health, and the WHO has established a global digital health strategy for the years 2020–2025 [1]. The overarching aim of the WHO’s global strategy is to improve population health through the use of digital technologies [1]. An estimated 8 out of 10 internet users seek health information online before consulting healthcare professionals [2]. Digital technologies are frequently utilized in the rehabilitation of musculoskeletal disorders [3] and previous studies have shown that physiotherapists tend to prefer real-time, video-based technology over telephone-based modalities, as it enables visual interaction [3, 4].

Osteoarthritis (OA) is one of the most prevalent musculoskeletal disorders worldwide, with substantial implications for individuals, healthcare systems, and socioeconomic costs [5, 6]. OA primarily affects the knees, hips, and hands, although other joints may also be involved [5]. In 2020, an estimated 595 million individuals were affected by OA globally, with knee osteoarthritis (KOA) accounting for approximately 62% of all cases [5]. The global prevalence of KOA is projected to reach 642 million by 2050 [5]. In Sweden, the prevalence of OA is projected to increase markedly; by 2032, 30% of individuals aged 45 years or older are expected to have OA, with half of these cases involving KOA [7]. The current prevalence of KOA in Sweden is approximately 14%, with risk increasing substantially with age—affecting 4% of individuals aged 45–54 years and more than 30% of those aged 85 years and older [7].

According to international guidelines, the assessment of KOA should be based on a thorough patient history and physical examination [8, 9]. KOA symptoms vary in severity, ranging from mild to severe, and may present as either intermittent or persistent [9]. Common complaints include activity-related knee pain and either an absence of joint-related morning stiffness or stiffness lasting less than 30 minutes [9]. Additional symptoms relevant for KOA assessment are persistent knee pain, limited morning stiffness, and functional impairments [8]. Guidelines emphasize the importance of a physical examination to establish an accurate diagnosis, with key clinical signs including crepitus, reduced range of motion, and bony enlargement [8]. A patient presenting with several positive clinical findings is highly likely to have KOA [8]. Radiography is not required for diagnosis, but may be indicated when alternative diagnoses are suspected [8–10]. While radiography can reveal structural changes consistent with KOA, symptoms often does not correlate with radiographic findings [8–10]. In Sweden, physiotherapists in primary care settings routinely diagnose KOA without radiographic imaging [11]. Physiotherapists have been suggested to be comparable to physicians as first-line assessors of KOA [12], and physiotherapy-led assessments may result in lower healthcare costs compared with physician-led assessments [13].

Several studies [14–16] on digital rehabilitation interventions for KOA have reported outcomes comparable to those of face-to-face rehabilitation and superior to those of self-directed care. A recent systematic review [17] of digital physiotherapy assessments for musculoskeletal disorders, compared with face-to-face assessments, demonstrated inconsistent findings regarding the validity and reliability of digital assessments. In addition, a smaller study [18] involving 18 participants with knee pain reported comparable validity and reliability between video-based and face-to-face assessments. Nonetheless, the current evidence on the validity and reliability of video-based assessment for KOA remains limited, underscoring the need for further, larger-scale studies in this field.

According to clinical guidelines, physical examination is a fundamental component of an adequate clinical assessment for diagnosing KOA [8]. However, when a physical examination is not feasible, it is important to investigate whether KOA can be assessed in a valid and reliable manner through video-based assessment. The purpose of this pilot study was to explore the feasibility of video-based physiotherapy assessment of KOA in patients with knee pain. Additionally, we aimed to provide preliminary data on its concurrent validity and interrater reliability compared with conventional face-to-face assessment.

## Method

### Study design

This cross-sectional methodological pilot study, conducted in June 2024, evaluated the concurrent validity and interrater reliability of video-based physiotherapy assessments. The study reporting followed the Consensus-based Standards for the Selection of Health Measurement Instruments (COSMIN) [19]. Concurrent validity [20] was assessed by comparing the agreement between video-based physiotherapy assessment and conventional face-to-face assessment for KOA. In this study, the diagnose KOA refers to a clinical diagnosis based on patient history and physical examination, as recommended in primary care guidelines [8, 9]; radiographic confirmation was not included in the protocol. Clinical assessment was performed by physiotherapists [12], and the conventional face-to-face assessment was considered the gold standard in this pilot study. Interrater reliability was evaluated by having a third physiotherapist independently review recordings of the video-based assessments.

The study was conducted in accordance with the ethical standards of the responsible institutional and national committees on human experimentation and with the Declaration of Helsinki of 1975, as revised in 2000 [21]. Ethical approval was obtained from the Swedish Ethical Review Authority (reference number: 2024-022 07 − 01).

### Participants

Participants were recruited from Region Västra Götaland through advertisements on social media and at a fitness center over a one-week period. The advertisements included a link to a declaration of interest and a web-based application form. Individuals who expressed interest were contacted by the first author (CH) to confirm eligibility according to the inclusion and exclusion criteria. The inclusion criteria were: age 45 years or older; a history of knee pain for at least four weeks; sufficient linguistic and cognitive ability to understand conversational Swedish; access to a videoconferencing device (e.g., smartphone, computer, or tablet); and ability to travel to the rehabilitation clinic in Gothenburg. Exclusion criteria were: (1) prior assessment by a physiotherapist or physician for the current knee pain; (2) arthroplasty of the knee or hip on the affected side; (3) ongoing treatment for the current knee pain; and (4) knee trauma within the past four weeks. All participants received oral and written information about the study, and written informed consent was obtained prior to participation.

### Sample size

A sample size calculation was performed using Power Analysis and Sample Size (PASS) for Windows, version 24.0.1 (NCSS, Kaysville, Utah, USA) based on Cohen’s kappa. The calculation indicated that 31 participants were required to detect an agreement of 0.6 with 80% power. Assumptions included an expected KOA prevalence of 30%, a null hypothesis value of 0.2, and an alternative hypothesis specifying a Cohen’s kappa coefficient (κ) not equal to 0.2, with an alpha level of 0.15. Given the pilot and feasibility design, the sample size was pragmatically determined to balance statistical considerations with available resources and timeframe. An alpha level of 0.15 was accepted to allow for a smaller sample, this significantly increases the risk of Type I error, and the findings should therefore be interpreted as exploratory. A total of 40 participants were recruited to account for an anticipated dropout of 9 participants (22.5%).

### Procedure

All participants underwent both video-based and conventional face-to-face assessments. The video-based assessments were recorded and subsequently evaluated by a second physiotherapist who had not been involved in conducting either the initial video-based or the conventional face-to-face assessments. In total, seven physiotherapists participated in the study. The physiotherapists’ mean age was 32 years, with professional experience ranging from 3.5 to 17 years and experience in primary care ranging from 2.5 to 17 years. Their academic qualifications ranged from a bachelor’s degree to a doctoral degree.

Four physiotherapists (three females and one male) conducted the video-based and conventional face-to-face assessments. An additional three physiotherapists (two females and one male) independently evaluated the recorded video-based assessments. Data collection was performed using a digital reporting form developed in esMaker, Version NX3-v3.0 (Entergate, Halmstad), based on established guidelines for KOA assessment [8, 9]. The form ensured that physiotherapists systematically captured key aspects of KOA assessment, with the primary outcome being the presence of KOA (yes/no), while also documenting secondary outcomes such as pain at rest and during movement, suspicion of red flags, adverse events, and referrals to other categories of care.

Assessments were conducted at the physiotherapists’ discretion and adapted based on the patients’ symptoms, in accordance with standard practice in Sweden. The reporting form allowed ample opportunity for written comments. Two pilot tests of the reporting form were conducted prior to the study, resulting in minor adjustments. To ensure consistency in its use, the physiotherapists performing the video-based and conventional assessments participated in a one-hour introduction to the study and the reporting form. The order of participants’ assessments was randomized using sealed opaque envelopes prepared by an individual independent of the study.

Real-time video-based assessments were conducted by a physiotherapist while participants were in an environment of their choice. During the assessment, the physiotherapist verified the patients’ current location (address) in case of an emergency. A conventional platform for real-time audio and video communication was used (Care Platform, version 3.27.9, Stockholm). To assess interrater reliability in this pilot study, the video-based assessments were recorded using Open Broadcaster Software for Mac and Windows, version 30.1.2 (OBS Studio Contributors).

The conventional face-to-face assessments were conducted at a rehabilitation clinic in Gothenburg. For each participant, the video-based and face-to-face assessments were performed during the same week, with each assessment lasting approximately 45 minutes. Participants were informed of their results following the completion of both assessments.

### Outcome measures

Concurrent validity was defined as the agreement between the video-based physiotherapy assessment and the conventional face-to-face assessment for diagnosing KOA (yes/no) in patients with knee pain. Interrater reliability was defined as the agreement between the video-based assessment and the recorded video-based assessment for diagnosing KOA (yes/no). The primary analysis focused on exact agreement, defined as identical diagnoses (KOA yes/no) between assessments. The secondary exploratory analysis examined potential agreement, which was defined as cases where the assessments did not match exactly but the physiotherapists’ notes suggested a possible diagnosis of KOA, such as indications of meniscus-related findings or other clinical signs suggestive of KOA. Notes provided by the assessing physiotherapists were manually analyzed based on clinical rationale in Sweden and previous research to identify additional similarities and the potential for KOA diagnosis. Meniscus-related findings are significantly associated with early stage of KOA development [22, 23], and individuals with meniscal degeneration have a fivefold higher risk of developing KOA within four years [24]. Therefore, notes referred to meniscus-related findings or indicating suspicion of KOA were reclassified as having potential KOA, reflecting this potential diagnosis.

Demographic data, including age, sex, height, weight, comorbidities, and previous experiences with digital care, were collected digitally from participants prior to the assessments. Body mass index (BMI) was calculated as weight (kg) divided by height squared (m²). Secondary outcomes, including suspicion of red flags, adverse events, and referrals to other categories of care, were presented descriptively. Self-reported pain at rest and during movement was assessed using the Numerical Rating Scale (NRS), with scores ranging from 0 (no pain) to 10 (worst imaginable pain) [25]. The NRS has demonstrated excellent test-retest reliability and validity for measuring pain intensity in individuals with KOA, with an intraclass correlation coefficient (ICC) of 0.95 [26].

### Data analysis

All data were analyzed using the Statistical Package for the Social Sciences (SPSS) for Mac, version 29.0.2.0 (IBM Corp., Armonk, NY, USA). To evaluate concurrent validity, KOA (yes/no) was coded as a dichotomous variable and analyzed using percentages, frequencies, sensitivity, specificity, and Cohen’s kappa (κ) with 95% confidence intervals (CIs). The κ values were interpreted as follows: <0.00, poor agreement; 0.00–0.20, slight agreement; 0.21–0.40, fair agreement; 0.41–0.60, moderate agreement; 0.61–0.80, substantial agreement; and 0.81–1.00, almost perfect agreement [27]. The same methods were applied to assess interrater reliability.

Demographic data and secondary outcomes were analyzed and presented descriptively using percentages, frequencies, means with standard deviations (SD), or medians with interquartile ranges (IQR) [28].

## Results

**Fig. 1:**
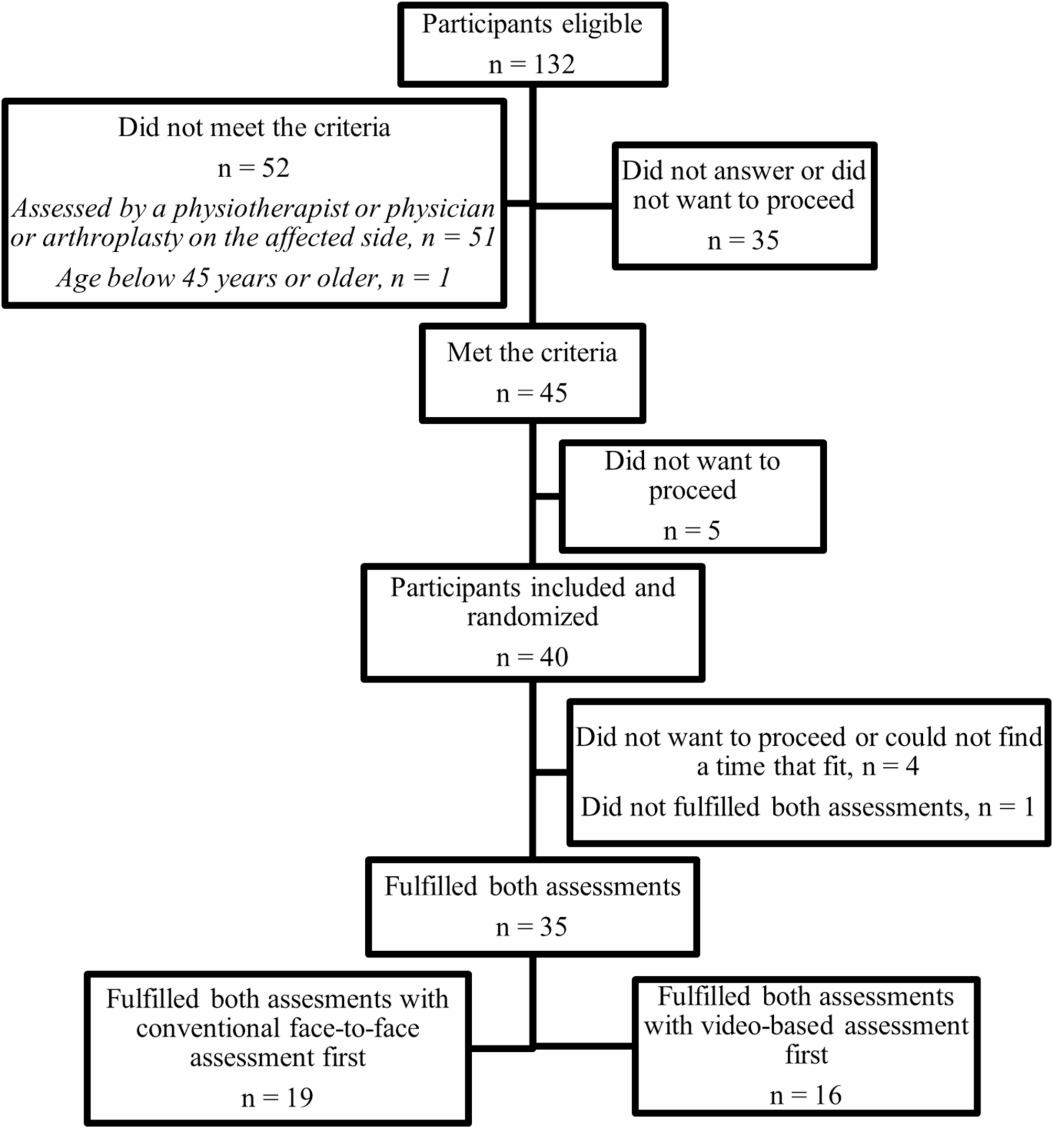
Flowchart of recruitment and inclusion in the pilot study

A total of 132 eligible participants expressed interest in the pilot study. Among these, 51 were excluded due to prior assessment by a physiotherapist or physician, or arthroplasty of the affected side; one participant was younger than 45 years; and 35 either did not respond or chose not to proceed. Overall, 45 participants (34% of those eligible) met the inclusion criteria. Of these, 40 participants were enrolled and randomized, with 35 completing both assessments. Sixteen participants completed the video-based assessment first, while 19 completed the conventional face-to-face assessment first. The inclusion and exclusion process is illustrated in Fig. 1.

### Participant characteristics

Thirty-five participants (17 females and 18 males) with a mean age of 64 years were included in the present pilot study. Among these participants, one had previously experienced digital care 6–20 times, while the remaining participants had experienced digital care 0–5 times. Nineteen participants (54%) reported no comorbidities, six participants (17%) had cardiovascular disease, two participants (5.7%) had joint disease, and eight participants (23%) reported other conditions, including asthma, hypothyroidism, cystic fibrosis, allergies and atopic eczema, type 2 diabetes, ulcerative colitis, chronic obstructive pulmonary disease, or prostate problems. Demographic characteristics of all included participants are presented in Table 1.

**Table 1:** Demographic data and pain at rest and during movement of all included participants

Participant demographics	Total, *n* = 35 (100%)
Age, years mean (SD)	64 (7.6)
Height, cm mean (SD)	176 (11.4)
Weight, kg mean (SD)	84 (21.0)
BMI, kg/cm^2^ mean (SD) Pain at rest, NRS at Face-to-face assessment, median [IQR] Pain at movement, NRS at Face-to-face assessment, median [IQR]	27 (4.4) 3 [1, 4] 5 [3, 6]

*BMI *Body Mass Index, *n* number of participants, *SD* Standard Deviation, *NRS* Numerical rating scale, *IQR* Interquartile range, *Kg* Kilogram

### Concurrent validity

The assessments for KOA (yes/no) demonstrated exact agreement in 28 of 35 cases (80%). Sensitivity was 83%, with 25 participants diagnosed with KOA, and specificity was 60%, with three participants classified as not having KOA. Cohen’s kappa indicated fair agreement, κ = 0.35 (95% CI: −0.03, 0.72). In the secondary exploratory analysis, which considered potential agreement, seven participants were reclassified as having potential KOA (Supplementary file 1). Overall concordance increased to 33 of 35 cases (94%). Sensitivity rose to 97%, with 31 participants diagnosed with KOA, and specificity was 67%, with 2 participants classified as not having KOA. Cohen’s kappa indicated substantial agreement, κ = 0.64 (95% CI: 0.17, 1.00). The reclassification provides some clinically relevant insight, but the secondary exploratory analysis cannot be considered confirmatory evidence of validity.

### Interrater reliability

Of the 35 video-based assessments, 29 were successfully recorded, while the remaining six were lost due to technical difficulties experiences by the physiotherapists with the recording software. Exact agreement of the assessments was observed in 25 of 29 cases (86%). Sensitivity was 92%, with 22 participants diagnosed with KOA, and specificity was 60%, with three participants classified a not having KOA. Cohen’s kappa was κ = 0.52 (95% CI: 0.10, 0.93), indicating moderate agreement. In the secondary exploratory analysis, which considered potential agreement, three participants were reclassified as having potential KOA (Supplementary file 1). Concordance increased to 27 of 29 cases (93%). Sensitivity was 96%, with 25 participants diagnosed with KOA, and specificity was 67%, with two participants classified as not having KOA. Cohen’s kappa indicated substantial agreement, κ = 0.63 (95% CI: 0.15, 1.00).

### Secondary outcomes

No acute adverse events occurred during either the video-based or the conventional face-to-face assessments. Minor technical problems were reported in ten of the video-based assessments, including hardware running out of power, software issues (e.g., entering the meeting, sound or video problems), and difficulties for participants in handling the equipment.

In the conventional face-to-face assessments, no participants were referred to another category of care by the physiotherapists. In the video-based assessments, two participants were referred to another category of care: one due to suspected psoriatic arthritis and the other due to suspected ulcerative colitis.

In the conventional face-to-face assessment, one participant was identified with a red flag based on their previous medical history. The same participant was not flagged in the video-based assessment, as no signs of fever, malaise, redness, discoloration, or increased local temperature were observed. In the video-based assessments, five participants were flagged with red flags, primarily due to their medical history (e.g., cancer or autoimmune conditions) and, in some cases, self-reported local heat increase. In these instances, the physiotherapists noted that no malaise or other concerning signs were observed during the conventional face-to-face assessments.

## Discussion

In this pilot study, video-based assessment showed indications of concurrent validity compared with conventional face-to-face assessment. Interrater reliability appeared moderate to substantial between the video-based assessment and the recorded assessment, suggesting that video-based assessment may be feasible for evaluating KOA in individuals with nontraumatic knee pain.

Concurrent validity and interrater reliability demonstrated greater agreement in the assessment of KOA compared with a previous study [18], which examined the agreement of knee pain regardless of diagnosis. This higher agreement in the present pilot study is not surprising, as it may be easier to identify and assess a single diagnosis compared with evaluating multiple diagnoses, which can introduce additional variability. Furthermore, the concurrent validity and interrater reliability observed here were similar to the agreement between the criteria for diagnosing KOA established by the American College of Rheumatology (ACR) and EULAR [29]. The ACR criteria showed a sensitivity of 95% and a specificity of 69% for KOA when at least three criteria were fulfilled [29]. These criteria are similar to those included in the reporting form used in this pilot study, where anamnesis plays a crucial role in establishing the diagnosis. Accurate diagnosis of KOA requires adequate patient history [8, 9], and the level of evidence is high for risk factors such as age over 50, female sex, and previous joint injury [8]. Additionally, the agreement observed for concurrent validity and interrater reliability in this pilot study is comparable to, or greater than, the agreement reported for physical examinations using common orthopedic tests [30]. Overall, the results indicate sufficient agreement in the assessment of KOA, suggesting that video-based assessment may be a feasible option.

Regarding the secondary outcomes, differences were observed in the assessment of red flags between the video-based and conventional face-to-face assessments. Physiotherapists appeared particularly attentive to red flags during the video-based assessments, possibly due to uncertainty about patient safety and concern about overlooking important signs. In video-based assessments, the physiotherapists had to rely on patients’ self-reports and could not directly evaluate, for example, the extent of swelling or heat increase. This finding aligns with previous studies [31, 32] reporting that practitioners often lack confidence in remote physical assessments and express concerns regarding patient safety. Nonetheless, the present study suggests that physiotherapists may have reasonable confidence in their ability to identify significant red flags when assessing KOA via video-based consultations in patients with knee pain.

No acute adverse events were reported during the study. During the video-based assessments, the physiotherapist confirmed each participant’s current location (address) to ensure preparedness in case of an emergency, in line with recommendations for digital physiotherapy [32]. The findings of this pilot study suggest that video-based assessment of patients with knee pain is safe and may serve as a useful component in the development of digital care.

This pilot study has several strengths. First, it is among the first to evaluate the concurrent validity and interrater reliability of video-based assessment in patients with knee pain, specifically investigating the assessment of KOA compared with conventional face-to-face assessment. Second, the physiotherapists involved had varied professional experience, academic degrees, and backgrounds in primary care. In clinical practice, physiotherapists often have diverse levels of experience and education, which supports the transferability of these results to real-world settings. Experienced physiotherapists in primary care are able to develop clinical reasoning and recognize diagnostic patterns [33]. In this study, although all physiotherapists had several years of professional and primary care experience, their prior exposure varied, reflecting the diversity commonly encountered in clinical practice.

Furthermore, a pragmatic study design was employed, considered relevant to ensure alignment with clinical practice [34]. In pragmatic trials, both external validity (generalizability) and internal validity (reliability or accuracy) are important [34]. External validity in this study was supported by having minimal exclusion criteria, making the findings transferable to clinical practice. Patients with knee pain represent a heterogeneous group, as those with KOA often present with comorbidities [35]. In this study, participants were not excluded due to the presence of comorbidities. Internal validity was strengthened through a randomization procedure designed to minimize potential learning effects between the two assessments [36]. Additionally, to further reduce this potential influence, the physiotherapists did not provide participants with any information about the assessments, and they were blinded to each other’s assessment results.

### Methodological considerations

Several limitations were identified in this pilot study. First, the assessments were conducted according to the physiotherapists’ discretion and preferences, varying depending on the patients’ symptoms, which introduced individual differences. Clinical reasoning develops over time [33]; therefore, differences in years of experience in primary care and academic training may have influenced the assessments. To support consistency, the physiotherapists used a reporting form to document clinical signs. However, the main question regarding KOA (yes/no) provided only a dichotomous outcome, which fails to capture nuances in diagnostic reasoning. A physiotherapist may be highly confident or only marginally confident in KOA diagnosis, yet both scenarios were coded identically. This loss of granularity reduces the sensitivity of interrater agreement statistics and may mask clinically meaningful differences. Some notes further indicated uncertainty regarding the confidence level required to establish a KOA diagnosis. Using an ordinal ratio scale [20] to quantify the physiotherapists´ confidence or adopting more structured criteria for KOA diagnosis, could provide additional insight and better capture variability. These limitations should be acknowledged, and the findings interpreted cautiously as exploratory rather than confirmatory.

Second, variations in the diagnostic criteria for KOA [8, 9, 29] contributed to a lack of consensus among the physiotherapists, as reflected in their reporting form notes. This variability partly resulted in inconsistencies in establishing the KOA diagnosis. To further explore this, a secondary exploratory analysis based on potential agreement was conducted, in which comments suggesting meniscal pathology were reclassified as KOA. This approach is justified given the strong association between meniscal changes and early-stage KOA development [22, 23]. However, the subjective nature of this reclassification necessitates cautions interpretation, and these findings cannot be considered confirmatory evidence of validity. Furthermore, reporting form permitted physiotherapists to provide notes with varying levels of detail, with some offering extensive information, while others providing minimal or no commentary. This variability introduced additional inconsistency into the secondary analysis. Implementing clearer guidelines in the reporting form for KOA assessment, alongside with more detailed instructions for note-taking, could have supported more standardized conditions.

A conventional platform for real-time audio and video in video-based assessment was used. Third, a conventional platform for real-time audio and video was used for the video-based assessments, and several technical issues were reported, which may have influenced the results. Improved technical conditions could have facilitated more comparable assessments between video-based and face-to-face methods. Previous studies have highlighted that technical challenges in video-based physiotherapy can cause frustration, increase uncertainty, and reduce trust for both patients and clinicians [37, 38]. Therefore, technical difficulties in this study cannot be ruled out as a factor that negatively affected the video-based assessment of KOA.

In this pilot study, several assumptions in the sample calculation were made based on practical considerations and were applied only for assessing concurrent validity, not interrater reliability. Based on previous studies [7, 39], it was assumed that 30% of participants would have KOA. The alpha level was set at 0.15 to allow for a smaller sample; however, this substantially increases the risk of Type I error, and thus the findings should be interpreted with caution and considered exploratory. A conventional alpha of 0.05 would indicate a stricter threshold. These considerations underscore the need for larger, confirmatory studies to validate the findings regarding both concurrent validity and interrater reliability.

### Clinical implications and directions for future research

Results from this pilot study indicate that video-based assessment might be a feasible method for physiotherapists evaluating KOA in patients over 45 years of age with nontraumatic knee pain. Given the exploratory nature and small sample of this study, the results should be interpreted cautiously. The study also highlights the variability in clinical assessment of KOA, suggesting a need for greater consensus among physiotherapists. Future research with larger samples is needed to further explore the validity and reliability of video-based assessments, investigate differential diagnoses, and examine patients’ experiences. Such work may help inform more standardized approaches to assessing KOA in primary care settings.

## Conclusions

Video-based physiotherapy assessment appears feasible and may provide preliminary indications of validity for diagnosing KOA in individuals with nontraumatic knee pain. The results suggest acceptable interrater agreement and highlight the need for more standardized digital assessment protocols to ensure consistent and reliable use in clinical practice.

## Supplementary Information

Supplementary Material 1.

## Data Availability

The data can be obtained from the corresponding author upon reasonable request. The data are stored in a controlled-access facility at Region Västra Götaland.

## Abbreviations

ACR: American College of Rheumatology
CH: Christin Heina
CMH: Chan-Mei Ho Henriksson
COSMIN: Consensus-based Standards for the Selection of Health Measurement Instruments
EL: Elvira Lange (corresponding author)
ICC: Intraclass correlation coefficient
IQR: Interquartile range
KOA: Knee osteoarthritis
NRS: Numerical Rating Scale
OA: Osteoarthritis
PASS: Power Analysis and Sample Size
SB: Susanne Beischer
SD: Standard deviation
SPSS: Statistical Package for Social Science
WHO: World Health Organization
