# Supplementary material for: Video-based physiotherapy assessment of knee osteoarthritis in patients with knee pain: a validity and reliability pilot study
Source: BMC Musculoskelet Disord. 2025 Sep 23;26:853. doi: 10.1186/s12891-025-09201-x (PMC12455796; doi:10.1186/s12891-025-09201-x)
Supplement: Supplementary file 1 — Supplementary Material 1. [file 12891_2025_9201_MOESM1_ESM.docx]

**Appendix:** Diagnosis at the assessments and reclassification.

| **Age** | **Sex** | **Conventional Face-to-face assessment, KOA or Not KOA** | **Video-based assessment, KOA or Not OA** | **Recorded video-based assessment, KOA or Not KOA** | **Reclassification, Face-to-face assessment** | **Reclassification, Video-based assessment** | **Reclassification, recorded video-based assessment** |
| --- | --- | --- | --- | --- | --- | --- | --- |
| 61 | F | Not KOA | KOA | KOA | Potential KOA | - | - |
| 54 | F | Not KOA | KOA | KOA | Potential KOA | - | - |
| 50 | M | Not KOA | KOA | KOA | Potential KOA | - | - |
| 67 | M | Not KOA | Not KOA | KOA | Potential KOA | Potential KOA | - |
| 76 | M | KOA | Not KOA | Not KOA | - | Potential KOA | Potential KOA |
| 62 | M | KOA | KOA | Not KOA | - | - | Potential KOA |
| 60 | M | Not KOA | KOA | NA | Potential KOA | - | - |
| 65 | F | KOA | Not KOA | KOA | - | - | - |
| 53 | M | KOA | KOA | Not KOA | - | - | - |
| 65 | M | Not KOA | KOA | NA | - | - | - |
| 68 | M | Not KOA | Not KOA | Not KOA | - | - | - |
| 61 | F | Not KOA | Not KOA | Not KOA | - | - | - |
| 65 | F | KOA | KOA | KOA | - | - | - |
| 69 | F | KOA | KOA | KOA | - | - | - |
| 75 | F | KOA | KOA | KOA | - | - | - |
| 65 | F | KOA | KOA | KOA | - | - | - |
| 65 | F | KOA | KOA | KOA | - | - | - |
| 58 | F | KOA | KOA | KOA | - | - | - |
| 65 | F | KOA | KOA | KOA | - | - | - |
| 56 | F | KOA | KOA | KOA | - | - | - |
| 66 | F | KOA | KOA | KOA | - | - | - |
| 61 | F | KOA | KOA | KOA | - | - | - |
| 72 | F | KOA | KOA | KOA | - | - | - |
| 75 | M | KOA | KOA | KOA | - | - | - |
| 75 | M | KOA | KOA | KOA | - | - | - |
| 65 | M | KOA | KOA | KOA | - | - | - |
| 67 | M | KOA | KOA | KOA | - | - | - |
| 69 | M | KOA | KOA | KOA | - | - | - |
| 62 | M | KOA | KOA | KOA | - | - | - |
| 57 | M | KOA | KOA | KOA | - | - | - |
| 77 | M | KOA | KOA | KOA | - | - | - |
| 68 | M | KOA | KOA | NA | - | - | - |
| 76 | F | KOA | KOA | NA | - | - | - |
| 51 | M | KOA | KOA | NA | - | - | - |
| 50 | F | KOA | KOA | NA | - | - | - |

*F; Female, M; Male, KOA; Knee osteoarthritis, NA = not available; indicates unrecorded data.*
